# Supplementary material for: Splice-Junction-Based Mapping of Alternative Isoforms in the Human Proteome
Source: Cell Rep. Author manuscript; Available in PMC 2020 Jan 15. (PMC6961840; doi:10.1016/j.celrep.2019.11.026)
Supplement: 2 [file NIHMS1546469-supplement-2.zip › DF1/PXD006675/TricuspidValve/TricuspidValve_1_SYPL1_ETSLHSPSNTSAPHSQGGIPPPTGL.pdf]

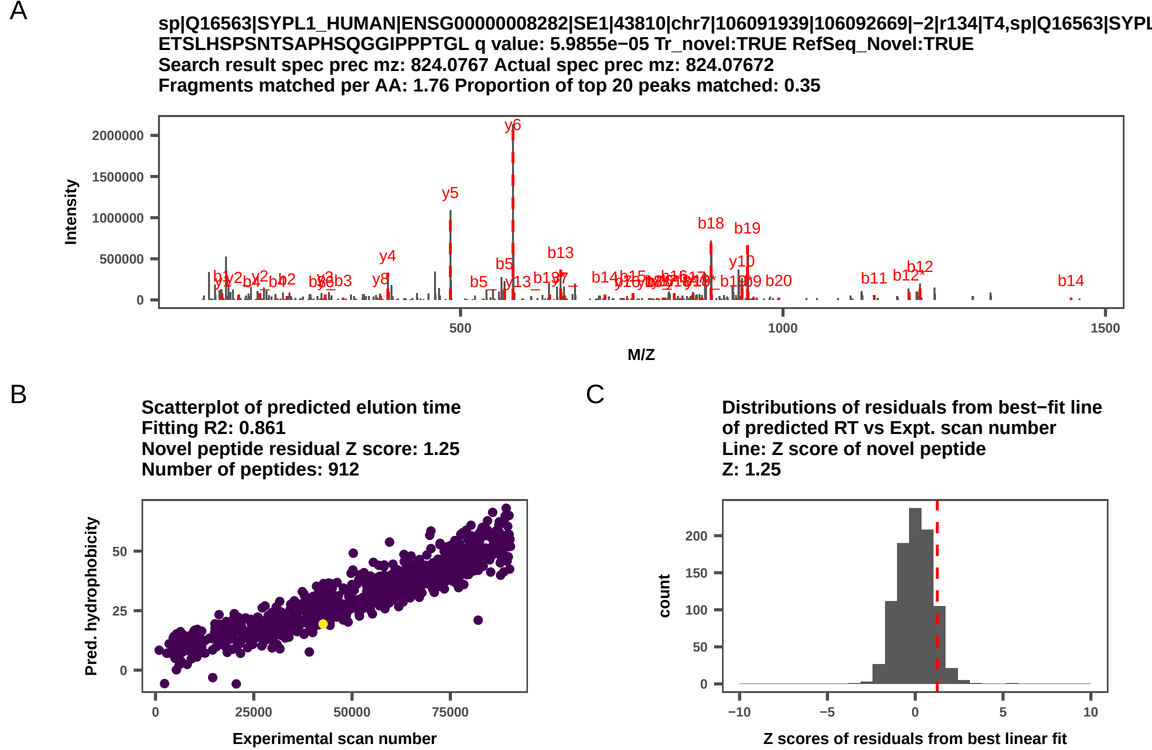
A
sp|Q16563|SYPL1_HUMAN|ENSG00000008282|SE1|43810|chr7|106091939|106092669|−2|r134|T4,sp|Q16563|SYPL
ETSLHSPSNTSAPHSQGGIPPPTGL q value: 5.9855e−05 Tr_novel:TRUE RefSeq_Novel:TRUE
Search result spec prec mz: 824.0767 Actual spec prec mz: 824.07672
Fragments matched per AA: 1.76 Proportion of top 20 peaks matched: 0.35
Intensity
2000000
1500000
1000000
500000
0
y6
y5
y4
b18
b19
b13
b5
y10
b20
b11
b12
b14
500
1000
1500
M/Z
B
Scatterplot of predicted elution time
Fitting R2: 0.861
Novel peptide residual Z score: 1.25
Number of peptides: 912
Pred. hydrophobicity
50
25
0
0
25000
50000
75000
Experimental scan number
C
Distributions of residuals from best−fit line
of predicted RT vs Expt. scan number
Line: Z score of novel peptide
Z: 1.25
count
200
150
100
50
0
−10
−5
0
5
10
Z scores of residuals from best linear fit
